# Prevalence of Pulmonary Tuberculosis among Adults in a North Indian District

**DOI:** 10.1371/journal.pone.0117363

**Published:** 2015-02-19

**Authors:** Ashutosh N. Aggarwal, Dheeraj Gupta, Ritesh Agarwal, Sunil Sethi, Jarnail S. Thakur, Sharada M. Anjinappa, Vineet K. Chadha, Rajesh Kumar, Meera Sharma, Digambar Behera, Surinder K. Jindal

**Affiliations:** 1 Department of Pulmonary Medicine, Postgraduate Institute of Medical Education and Research, Chandigarh, India; 2 Department of Medical Microbiology, Postgraduate Institute of Medical Education and Research, Chandigarh, India; 3 School of Public Health, Postgraduate Institute of Medical Education and Research, Chandigarh, India; 4 Epidemiology and Research Division, National Tuberculosis Institute, Bangalore, India; Hopital Raymond Poincare - Universite Versailles St. Quentin, FRANCE

## Abstract

**Background:**

Recent population prevalence estimates of pulmonary tuberculosis (PTB) are not available for several areas in India. We conducted a field-based population survey at a north Indian district to estimate point prevalence of bacteriologically positive PTB.

**Methods:**

A stratified cluster sampling design was used to conduct the survey in both urban and rural areas within the district. All adults aged more than 15 years, in 18 rural and 12 urban clusters of 3000 subjects each, were interviewed using a symptom card. Two sputum samples were collected from all persons having symptoms suggestive of PTB, or history of antitubercular treatment, for smear microscopy for acid-fast bacilli and mycobacterial culture. Those having at least one sputum specimen positive on microscopy and/or culture were categorized as having PTB. Prevalence was estimated after adjusting for cluster sampling and incomplete data (through individual level analysis with robust standard error).

**Results:**

Of 91,030 eligible adult participants (47,714 men and 43,316 women), 85,770 (94.2%) completed the symptom cards. Of them, 2,898 persons were considered eligible for sputum examination and 2,839 (98.0%) provided at least one sample. Overall, 21 persons had bacteriologically positive PTB, and cluster level prevalence was estimated at 24.5 per 100,000 population (95% CI 12.8–36.2). Individual level analysis with robust standard error yielded a prevalence estimate of 24.1 per 100,000 populations (95% CI 12.8–35.4).

**Conclusion:**

The observed prevalence of bacteriologically positive PTB in this district is lower than empiric national estimates, probably as a result of successful implementation of tuberculosis control measures in the area.

## Introduction

Tuberculosis (TB) prevalence surveys are important in assessing the performance of TB control programs, providing information for planning, and assessing trends of the disease burden over time. This is particularly important in India where several patients receive treatment from practitioners not part of the national disease control programme, and hence notification data reported through the programme are insufficient to reflect the overall burden of disease.

A nation-wide sample survey conducted during 1955–1958 estimated that India had a high prevalence of TB (400 microbiologically confirmed pulmonary TB cases, and 16,000 TB suspects with chest radiographic abnormalities, per 100,000 population) [1]. Extrapolation of these findings, immediately prior to initiation of the Revised National Tuberculosis Control Programme (RNTCP), in 1997 estimated the national burden to be around 3.5 million confirmed pulmonary TB cases and 14 million radiologically abnormal TB suspects [2]. Subsequently, in 2005, an Expert Committee revised the estimated national burden to be around 8.5 million cases (inclusive of microbiologically proven, radiologically suspected, and extrapulmonary TB patients) based on a few studies in small geographic locations [3]. The RNTCP has been quite successful in achieving high case detection and treatment rates in India, and data from isolated centers suggest a reduction of TB prevalence of upto 50% or more with successful implementation of this programme [4,5]. In this context, a need was felt to gather fresh prevalence estimates in a scientific manner at multiple locations across the country. Since nationwide surveys are neither economical nor administratively feasible, small regional surveys at sentinel sites on a sustainable basis over the next several years were planned. The Government of India identified six institutions to conduct prevalence surveys at seven sites using a generic protocol, with an aim to assess the point prevalence of pulmonary TB in the adult community at each site. We herein report the results from the population survey conducted in Sahibzada Ajit Singh Nagar, which is part of the Mohali district of state of Punjab in North India.

## Subjects and Methods

This cross-sectional study used a common protocol used by all sites involved in the project. Results of the surveys conducted at a rural area alone near Bangalore in southern India, and urban and rural areas of Jabalpur in central India, have already been reported [5,6].

### Sampling

Previous TB prevalence figures for the population residing in the district of Mohali were not available. The expected national prevalence of bacteriologically proven pulmonary TB was arbitrarily considered to be 240 per 100,000 adult population, based on expert estimates. Sample size was calculated as 79,840 to estimate the prevalence within 20% of this expected estimate at 5% significance level, and a arbitrarily chosen design effect of 2.0 to account for clustering. To adjust for a final expected survey coverage rate of about 90%, we decided to study 90,000 individuals. It was planned to conduct the study in 30 clusters of approximately 3,000 subjects each.

A listing of rural villages and urban municipal wards, as well as population statistics, was obtained from the Directorate of Census Operations in the state. Based on the last available census data at time of survey, the population of Mohali district was estimated at 698,317, with 427,044 (61.2%) and 271,273 (38.8%) persons living in rural and urban areas respectively. Based on this rural-urban distribution of population in the district, we decided to target 54,000 (60%) residents in 18 rural clusters, and 36,000 (40%) residents in 12 urban clusters. The survey sites chosen for the study were identified through random sampling from a listing of all villages (for rural areas) and municipal wards (for urban areas). In case the adult population of a village was substantially less than 3,000, the survey was extended to an adjoining village, identified from the local map, till the required cluster sample size was achieved.

### Survey methodology

The field survey was carried out from July 2008 to March 2010. The survey team leader made an initial visit to the site of the survey to meet local officials, village heads, and others to seek their cooperation and collect information regarding population, approximate number of households, lanes and hamlets, and local healthcare facilities. In rural areas, a short note on the purpose and description of the workplan was provided to village leaders in local language, for announcements to be made at public and religious meeting places.

The field survey staff was broadly divided into two groups—the enumerator team and the sputum collection team, working parallelly in the same area. Within each of the identified clusters, field enumerators interviewed all adult members (aged more than 15 years) of each household. Visitors temporarily residing in the households were not interviewed. Interviews were conducted face to face, in privacy and inside the homes of the respondents. In case a household was locked or a respondent not available, the field worker returned on a subsequent day at a mutually convenient time to complete the interview. If two such attempts at meeting residents of a household were unsuccessful, the household was dropped from the list.

Each respondent received a unique study identifier number. The TPT69 (Tuberculosis Prevention Trial 69) card, developed and extensively used by the Tuberculosis Research Centre, Chennai, was used to collect basic demographic data, current pulmonary symptoms, and past history suggestive of pulmonary tuberculosis. The card has 29 response boxes, each with a unique set of response codes. In addition, we also collected information regarding current and past use of antitubercular therapy, and results of sputum microbiology for the relevant episodes (if known). As part of internal quality control, the team leader re-interviewed 10% randomly selected persons on the same day. Any respondent having (a) persistent cough for two or more weeks, (b) fever for one month or more, (c) chest pain for one month or more, (d) hemoptysis within the past six months, or (e) history of previous treatment for TB was eligible for sputum examination. Sputum samples were obtained by the sputum collection team as per a uniform protocol (see details below). Tuberculin skin testing or chest radiography was not performed.

Prior to start of the survey, all field staff received centralized training at the Tuberculosis Research Centre, Chennai. Training included modules on door-to-door census and registration, elicitation of symptoms, sputum collection, and data management. To ensure that the quality of data collected was uniform, all field workers were instructed to follow all instructions carefully and exactly, so that methodology used by all interviewers was identical. Questions were asked exactly in the sequence in which they were printed in the questionnaire. Each question was read aloud exactly as written, without altering the wording. If the question was not understood, the interviewer could use additional explanations or examples provided in instructions for individual questions in the project manual. If no additional instructions were provided in the manual, the question was repeated in its original form, without probing for an answer. If, even after a brief explanation, doubt remained as to whether the answer to a particular question was ‘Yes’ or ‘No’, the response was recorded as ‘No’. However, interviewers could listen to additional comments from the respondents as this helped in improving rapport.

### Sputum collection, processing and microbiology

The sputum collection team collected a spot sputum sample from eligible respondents in sterile plastic containers, on the same day as the primary interview. Simultaneously, another container was left with the respondent to collect another sample early morning the next day. This sample was picked up by the sputum collection team on their visit to the field area the next day. All sputum samples were carefully marked with the respondent’s identifier number and timing of sample (spot or early morning) and transported to the Mycobacteriology Laboratory of our institute on the same day.

Sputum smears were prepared directly from each sputum specimen, and also from concentrated samples. Smears were stained using Ziehl-Neelsen stain and examined with oil-immersion microscopy for the presence of acid-fast bacilli (AFB). About ten percent of randomly selected sputum smears were cross-examined by one of the investigators (SS), and quality assurance protocols were followed as per RNTCP guidelines. For sputum culture, sputum samples were decontaminated by N-acetyl-L-cysteine-sodium hydroxide (NALC-NaOH) method and inoculated on two slopes of Lowenstein-Jensen (LJ) medium. Cultures were incubated at 37°C for eight weeks and examined every week for growth of mycobacterial colonies. Any suspected growth was confirmed by Ziehl-Neelsen staining for AFB, and subcultured on fresh LJ media for further identification. Culture growth was confirmed as *Mycobacterium tuberculosis* using standard morphologic parameters (growth rate, colony morphology and colour) and biochemical reactions (niacin production, nitrate reduction, loss of catalase activity at 68°C, and susceptibility on LJ medium containing para-nitrobenzoic acid) [7]. The cultures were discarded in case of any contamination or lack of growth by eight weeks.

### Data handling and analysis

A daily work report was prepared by the field staff. Reported numbers for interviews conducted and sputum samples collected were cross-checked every evening with the actual questionnaire forms and sputum samples submitted. An independent data entry team checked all questionnaire forms for accuracy and completion, and transcribed the information into a computer database designed specifically for this study.

Any individual having at least one sputum sample showing acid fast bacilli on direct smear microscopy, irrespective of sputum culture result, was considered a ‘smear positive case’. Any individual having at least one sputum culture showing growth of *Mycobacterium tuberculosis*, irrespective of sputum smear result, was considered a ‘culture positive case’. Any individual having at least one sputum sample showing acid fast bacilli on direct smear microscopy and/or growth of *Mycobacterium tuberculosis* was considered a ‘bacteriologically positive case’. Crude prevalence of pulmonary TB was estimated by dividing the number of bacteriologically positive cases by the total eligible study population actually surveyed. These crude estimates were adjusted to account for clustered sampling to derive a final cluster level prevalence estimate. Average prevalence across clusters (P_cluster_) was calculated as ∑P_i_/C, where P_i_ is the crude prevalence for the i^th^ cluster and C is the number of clusters (30 for this study). Standard error (SE) was calculated as SD/√C where SD = √[∑(P_i_−P_cluster_)^2^/C(C-1)]. Confidence intervals (95%) were calculated as the mean prevalence across clusters ± 1.96SE. Both crude and cluster level prevalence was based on information from only those persons for whom all actual data was available for analysis. Additionally, to account for bias introduced by incomplete data, between-cluster variability, and uncertainty in estimating SE, individual level analysis was conducted using logistic regression modeling with robust standard error, as previously described [5]. This approach assumed that data are missing at random within groups of respondents belonging to the same gender, age band, and symptoms suggestive of TB.

### Ethical considerations

The study protocol was approved by the Institute Ethics Committee of Postgraduate Institute of Medical Education and Research, Chandigarh. A written informed consent was obtained from each eligible participant aged 18 years or above, and from parent (or legal guardian) of participants aged 15–18 years, prior to inclusion into the study. In case a study participant was diagnosed to have pulmonary TB based on microbiological investigations carried out a part of the study, he/she was appropriately counseled, and directly observed antitubercular treatment facilitated through the nearest RNTCP center. Other individuals with respiratory symptoms (but not diagnosed as having TB) were counseled to seek medical advice at local healthcare facility, in case they had not already done so.

## Results

We surveyed 91,030 eligible adults (47,714 men and 43,316 women), of whom 54,726 (60.1%) were residents of rural areas. More than half the surveyed population in both rural and urban areas was aged less than 35 years (Table 1). Symptom cards were filled in 85770 (94.2%) individuals (Table 1). Agreement between paired data from 8062 individuals interviewed twice, as assessed by kappa estimate) was good for history of cough (0.8335), past TB treatment (0.8309) and eligibility for sputum evaluation (0.8678).

**Table 1:** Age and gender distribution of study population.

	Men		Women		Total	
	Number surveyed	Symptom cards filled	Number surveyed	Symptom cards filled	Number surveyed	Symptom cards filled
Total	47714 (100%)	43517 (91.2%)	43316 (100%)	42253 (97.5%)	91030 (100%)	85770 (94.2%)
Residence						
Rural	28988 (60.8%)	26463 (91.3%)	25378 (58.6%)	25146 (97.7%)	54726 (60.1%)	51609 (94.3%)
Urban	18726 (39.2%)	17054 (91.1%)	17578 (40.6%)	17107 (97.3%)	36304 (39.9%)	34161 (94.1%)
Age range (years)						
15–24	13998 (29.3%)	13511 (96.5%)	12327 (28.5%)	12109 (98.2%)	26325 (28.9%)	25620 (97.3%)
25–34	10808 (22.7%)	9975 (92.3%)	10660 (24.6%)	10447 (98.0%)	21468 (23.6%)	20422 (95.1%)
35–44	8609 (18.0%)	7646 (88.8%)	8035 (18.5%)	7829 (97.4%)	16644 (18.3%)	15475 (93.0%)
45–54	6347 (13.3%)	5487 (86.5%)	5276 (12.2%)	5129 (97.2%)	11623 (12.8%)	10616 (91.3%)
55–64	3895 (8.2%)	3379 (86.8%)	3802 (8.8%)	3672 (96.6%)	7697 (8.5%)	7051 (91.6%)
65–74	2753 (5.8%)	2390 (86.8%)	2222 (5.1%)	2129 (95.8%)	4975 (5.5%)	4519 (90.8%)
> = 75	1252 (2.6%)	1118 (89.3%)	966 (2.2%)	924 (95.7%)	2218 (2.4%)	2042 (92.1%)
Missing	52 (0.1%)	11 (21.2%)	28 (0.1%)	14 (50.0%)	80 (0.1%)	25 (31.3%)

Based on the survey criteria, 2898 persons (3.4% of the total population actually surveyed) were considered eligible for sputum examination. Among them, 2821 (97.3%) provided at least one sputum sample for microbiological investigations. 18 subjects (0.6%) provided only a spot sample, and 59 (2.0%) did not provide any sample. Reasons for not providing sputum samples included inability to cough out any sputum (for instance in persons with previous history of tuberculosis but no current respiratory symptoms), refusal to give sputum for examination, and inability of the sputum collection team to track down the concerned individual in time for sputum collection or sample retrieval.

Four subjects were positive on both smear and/or culture, of whom three showed such positivity on both spot and overnight samples. In addition, 17 subjects had negative sputum smears, but showed growth of *Mycobacterium tuberculosis* on culture either on spot sample (six subjects) or overnight sample (11 subjects). Thus the sensitivity of sputum smear examination in identifying persons with active pulmonary tuberculosis was quite low (19.0%) with solid culture as gold standard. Seven samples (six overnight and one spot) grew atypical mycobacteria on culture. In addition 235 of 5596 (4.2%) cultures showed contamination in one of the two slopes. For all these, the paired culture, as well as the culture from the other day, did not show mycobacterial growth. Thus 31 samples from 28 patients were positive on either smear and/or culture of which seven were finally identified as non-tubercular mycobacteria. Overall 21 samples grew *Mycobacterium tuberculosis* on culture. All samples showing mycobacteria on direct smear were also culture positive. Hence 21 respondents were diagnosed as bacteriologic cases and were used to estimate population prevalence of TB. Crude population prevalence was estimated at 24.5 per 100,000 adult individuals (95% CI 14.6–34.4). The crude population prevalence based only on smear positivity was much lower at 4.7 per 100,000 adult individuals (95% CI 0.09–9.23). Prevalence in rural areas (32.9 per 100,000, 95% CI 18.3–47.5) was higher than that in urban areas (11.7 per 100,000, 95% CI 0.9–22.5) and among men (34.5 per 100,000, 95% CI 18.2–50.8) as compared to women (14.2 per 100,000, 95% CI 3.6–24.8).

Overall cluster level prevalence was estimated at 24.5 per 100,000 population (95% CI 12.8–36.2). Individual level analysis with robust standard error yielded a prevalence estimate of 24.1 per 100,000 population (95% CI 12.8–35.4).

In addition, 59 individuals admitted to be taking antitubercular treatment at the time of survey, of whom 41 were receiving directly observed short course treatment (DOTS) at neighboring RNTCP facilities. Pre-treatment sputum examination had showed acid-fast bacilli for all these 41 subjects; sputum status of other 18 individuals was not known. All these 59 persons were part of the ‘sputum eligible’ population; however, none showed mycobacteria either on smear or culture during the present survey.

## Discussion

According to the recent WHO Global Tuberculosis Report, India accounts for nearly a quarter of the global TB burden, with approximately 2.8 million prevalent cases (prevalence rate 230 per 100,000) and 2.2 million incident cases every year [8]. Although numerically large in absolute terms, these indicators are better than those reported for previous years. This is largely attributed to a well-functioning national program to control the disease. Nationwide coverage of the RNTCP was achieved in 2006, and the program has shown consistent performance on key performance indicators ever since. Treatment success rates have more than tripled from 25% to 88%, and TB death rates have nearly halved, as compared to the pre-RNTCP era [9]. Overall strengthening of TB control activities is likely to have resulted in a reduction of TB prevalence in India.

This survey was part of a larger initiative by the Government of India to estimate TB prevalence in the background of a successful ongoing nationwide disease control programme. Results of a similar survey conducted in two other sentinel sites have already been reported, and have shown a relatively higher TB prevalence compared to what we estimated in this study. A survey of 99,918 adults in urban and rural areas in Jabalpur in central India showed an overall prevalence of 255.3 bacteriologically positive cases per 100,000 population [6]. Another survey on 71,874 adults in rural Bangalore in southern India showed an overall prevalence of 196 bacteriologically positive cases per 100,000 population [5]. Several factors could have contributed to the lower prevalence observed in our study, compared to estimates from other sites. In particular, the population to doctor ratio in Mohali district is quite good, with approximately one certified medical doctor for every 2000 population. It is likely that symptomatic individuals seek medical advice much earlier (and get treated faster), as compared to other geographic locations in India, lowering disease figures detected through surveillance data. The respiratory symptom profile of the population studied was also quite healthy, with only 3.4% of the population actually surveyed being eligible for sputum examination. In contrast, 8.3% and 7.4% individuals were eligible for sputum examination in the surveys conducted at Jabalpur and Bangalore respectively. Mohali is a satellite town of Chandigarh, the location of our institute, and the overall lower symptom prevalence in the study population is in line with our earlier estimates at Chandigarh. In an earlier survey conducted at urban Chandigarh and adjoining rural areas to estimate asthma prevalence using the 14-item International Union Against Tuberculosis and Lung Diseases symptom questionnaire, we found an overall prevalence of any respiratory symptom in only 4.3% and 4.7% of rural and urban respondents [10]. This was much lower than the symptom prevalence found at other centers in Delhi, Kanpur, and Bangalore. It is also possible that regional differences in TB prevalence and performance of RNTCP might have played a role in the lower observed prevalence. The third National Family Health Survey (2005–2006) reported wide variations in self-reported or medically treated TB across study samples from different states in India; the figure for Punjab (the state where Mohali is located) was 201 per 100,000 population as compared to a national average of 418 per 100,000 population [11]. It must, however, be noted that the methodology of this survey was not as robust, and the definition of TB used was rather imprecise and included all forms of TB, which is likely to have inflated these prevalence estimates. The overall educational and socio-economic status of residents in the district studied is good, and health personnel and healthcare infrastructure is better than most parts of the country [11]. This translates into health indicators (such as infant/child mortality rates and nutritional status) being better than the national average, and a lower prevalence of TB is not entirely unexpected.

The main strength of the study was a scientifically sound methodology that was uniformly applied to various survey locations across the country. The survey and microbiological analyses were carried out strictly as per the standard methodology. Strict reporting, recording, and quality control protocols were maintained. Despite this, there were some limitations. The biggest concern relates to the expert opinion for TB prevalence in India, which was used for sample size calculation. In the absence of a nationally valid figure, or recent estimates for the various regions surveyed, this is at best a single guesstimate that was used for all study sites. In retrospect, our study seems grossly underpowered in view of the substantial difference between prevalence figures used for sample size calculation and actual observed prevalence (240 per 100,000 population and 27.9 per 100,000 population respectively). The survey aimed at picking up additional TB cases, which were either presumably missed by the health system or had recently been started on treatment (and were therefore still bacteriologically positive). Such active case finding may often give imprecise estimates. This, along with the fact that the overall culture positivity rate was quite low, may have partly contributed to the lower sputum smear sensitivity observed in our study. Only 19.0% persons having positive sputum cultures showed AFB on sputum smears. In general, sputum smear examination identifies nearly half of all patients ultimately showing mycobacterial growth on solid culture [5,6]. It is possible that the ‘active’ nature of this survey picked up TB only in patients with quite early disease stage, as other more symptomatic patients are likely to have approached the health services earlier. However, since our microbiologic definition included culture results, we are unlikely to have missed patients in the final analysis. These finding underline the fact sputum microscopy alone is likely to miss a substantial proportion of active disease in the community in a survey of this nature. We also deliberately excluded patients on anti-tubercular therapy from prevalence calculations if they were bacteriologically negative during the survey. Such patients in a way reflect ‘period-prevalence’ of TB, and we felt it was improper to pool these patients with those identified during this survey directed at estimating ‘point prevalence’ of disease. Further, the goal of the study was to detect microbiologically proven TB, and not probable TB. Many other subgroups of patients (e.g. those with chest radiographic abnormalities, chronic respiratory symptoms, etc.) would also qualify to get designated as probable TB.

In conclusion, we estimated the population prevalence of bacteriologically positive pulmonary tuberculosis, at Sahibzada Ajit Singh Nagar at Mohali district in North India, as 24.1 per 100,000 adult population. Such a low figure in comparison to national estimates might be attributed to better health infrastructure at the study location, and the successful implementation of RNTCP in the district. Further surveys in the future would help us evaluate if this positive trend continues in this area.
